# Synergistic Strategies in Prostate Cancer Therapy: Electrochemotherapy and Electromagnetic Hyperthermia

**DOI:** 10.3390/pharmaceutics16091109

**Published:** 2024-08-23

**Authors:** Sayma Vizcarra-Ramos, Andrea Molina-Pineda, Abel Gutiérrez-Ortega, Sara E. Herrera-Rodríguez, Adriana Aguilar-Lemarroy, Luis F. Jave-Suárez, Zaira López, Mario E. Cano, Rodolfo Hernández-Gutiérrez

**Affiliations:** 1 Centro de Investigación y Asistencia en Tecnología y Diseño del Estado de Jalisco, A.C. (CIATEJ), Guadalajara 44270, Mexico; sayvimay0510@gmail.com (S.V.-R.); aortega@ciatej.mx (A.G.-O.); sherrera@ciatej.mx (S.E.H.-R.); 2 Centro de Investigación Biomédica de Occidente (CIBO), División de Inmunología, Instituto Mexicano del Seguro Social (IMSS), Guadalajara 44340, Mexico; adry.aguilar.lemarroy@gmail.com (A.A.-L.); lfjave@gmail.com (L.F.J.-S.); 3 Centro Universitario de la Ciénega, Universidad de Guadalajara, Avenida Universidad 1115, Ocotlan 47810, Mexico; zaira.lopez@academicos.udg.mx (Z.L.); mario.cano@academicos.udg.mx (M.E.C.)

**Keywords:** electrochemotherapy, bleomycin, cisplatin, electromagnetic hyperthermia, SPION, synergy

## Abstract

Prostate cancer is a significant global health problem, being the second most common cancer and the fifth leading cause of death in men worldwide. Standard chemotherapy, though effective, often lacks selectivity for tumor cells, resulting in dose-limiting side effects. To address this, innovative biomedical approaches such as electrochemotherapy and electromagnetic hyperthermia have emerged. Electrochemotherapy improves drug delivery by facilitating electroporation, thereby increasing intracellular concentrations of chemotherapeutic agents. This approach reduces dosages and associated adverse effects. Meanwhile, electromagnetic hyperthermia raises the temperature of tumor cells, enhancing their sensitivity to chemotherapy. While previous research has demonstrated the inhibitory effects of magnetic hyperthermia on prostate cancer cell growth both in vitro and in vivo, and its synergy with chemotherapy has shown enhanced tumor remission, limited studies have focused on electrochemotherapy alone or in combination with hyperthermia in prostate cancer models. This study aims to assess the synergistic effects of electromagnetic hyperthermia, with superparamagnetic iron oxide nanoparticles (SPIONs) and electrochemotherapy, with electroporation and the chemotherapeutic drugs bleomycin and cisplatin, on the prostate cancer-derived cell line DU-145/GFP and prostate-derived cell line RWPE-1. Results indicate enhanced cytotoxicity with both treatments (bleomycin and cisplatin) by adding electroporation, demonstrating a particularly pronounced effect with bleomycin. Combining electroporation with hyperthermia significantly augments cytotoxicity. Moreover, electroporation effectively reduced the time of exposure to electromagnetic hyperthermia while magnifying its cytotoxic effects. Future research in in vivo trials may reveal additional insights into the combined effects of these therapies.

## 1. Introduction

Prostate cancer is the second most common cancer worldwide and the fifth leading cause of death among males, with approximately 1.4 million new cases and 397,000 deaths estimated in 2022 [1]. The disease follows a gradual progression and requires a spectrum of treatments that vary by risk level [2,3]. Localized cancer can be treated with radical prostatectomy (RP) or radiation therapy (RT), improving survival but affecting urinary and erectile functions [3]. RT options include brachytherapy and external beam therapy, with possible side effects like cystitis and a small risk of secondary malignancies [4,5]. Ablative therapies such as high-intensity focused ultrasound (HIFU) and cryotherapy are used for low- to intermediate-risk cancer [4]. High-risk or advanced cancer is treated with androgen deprivation therapy (ADT) to slow tumor growth until castration-resistant disease appears, sometimes combined with RT or pre-RP [3,4,6]. Chemotherapeutic agents such as docetaxel and cabazitaxel are effective for treating advanced stages marked by metastasis and resistance to androgen deprivation therapy [7]. Nonetheless, their non-specific mechanism of action results in adverse effects including neuropathy, neutropenia, and pulmonary toxicity [8,9].

Nanotechnology holds immense potential for revolutionizing drug delivery in prostate cancer treatment. For example, nanoparticles can be engineered to deliver drugs with high precision directly to cancer cells, thereby minimizing side effects and enhancing therapeutic efficacy. Superparamagnetic iron oxide nanoparticles (SPIONs), for instance, have shown remarkable promise in this field. These nanoparticles can be functionalized to carry therapeutic agents and guided to tumor sites using external magnetic fields. This targeted approach not only improves drug accumulation at the tumor site but also allows for the controlled release of the drug, increasing its effectiveness while reducing systemic exposure. Additionally, emerging technologies such as electrochemotherapy enhance drug selectivity. This technique, which utilizes electroporation, facilitates the uptake of low-permeability chemotherapeutic drugs such as bleomycin and cisplatin. It increases intracellular drug concentration and cytotoxicity while minimizing side effects [10,11]. Research on electrochemotherapy (ECT) for prostate cancer is limited but promising. ECT, whether using bleomycin (BLM) or cisplatin (CDDP), induces immunogenic cell death and the release of damage-associated molecular pattern molecules. This, in turn, can strongly prime local cancer immunity and counteract tumor escape mechanisms. A 2008 preclinical study demonstrated that electrochemotherapy with bleomycin effectively suppressed tumor growth in mice, suggesting its potential for androgen-independent and resistant localized prostate cancer [12]. In 2017, Klein et al. reported a clinical case where electrochemotherapy using bleomycin and electroporation effectively removed a tumor in a patient with locally advanced prostate cancer, showing low toxicity and mild side effects [13].

Electroporation is also used in nanomedicine to deliver nanoparticles into cells [14]. Superparamagnetic iron oxide nanoparticles (SPIONs) represent a promising avenue, leveraging their magnetic properties to induce hyperthermia when exposed to alternating magnetic fields [15]. Magnetic hyperthermia raises the temperature to 40–45 °C, which can induce cell death through different mechanisms involving the rising temperature and the SPIONs themselves. 

Temperature primarily influences cell stability or damage during hyperthermia. Temperatures within 40–42 °C cause non-cytotoxic effects, including protein conformational changes, DNA synthesis inhibition, and heat shock protein expression that can induce the production of reactive oxygen species (ROS) and intrinsic apoptosis or activate survival mechanisms depending on their expression levels. Temperatures above 43 °C cause irreversible protein damage, leading to exponential cell death by apoptosis or necrosis [16,17]. Moreover, SPIONs can activate cell death via intrinsic or extrinsic pathways depending on their concentration; high concentrations activate caspase-8, while lower concentrations increase Bax and decrease Bcl-2 expression [18]. SPIONs alone can produce ROS through mechanisms involving iron ion release from lysosomal degradation, catalysis by nanoparticle surfaces, and mitochondrial and NADPH oxidase interactions [19,20]. Higher SPION concentrations elevate ROS production, leading to cell death, although low concentrations allow antioxidant defenses [19,21]. When irradiated with an electromagnetic field, SPIONs cause lysosomal membrane permeabilization, releasing enzymes that lead to necrosis or apoptosis depending on the extent of damage, facilitated by ROS production [19,22]. Magnetic hyperthermia, in addition to inducing cell death [23,24], can potentially augment chemotherapy efficacy by enhancing cellular sensitivity to drugs in vivo [25]. 

The preclinical evaluation of new treatments requires in vitro assays which are the initial stage for evaluating novel drugs due to their simplicity, ease of handling, and reproducibility [26], and are commonly performed on immortalized prostate cancer cell lines such as DU-145, representing advanced stages of the disease [27]. However, in vitro models lack the complexity of the tumor microenvironment, leading to less accurate representations of prostate cancer behavior than those seen in vivo [26].

While studies have demonstrated the inhibitory effects of magnetic hyperthermia on prostate cancer cell growth in vitro and in vivo [28,29] and its synergy with chemotherapy enhances tumor remission [30], limited research exists on the effects of electrochemotherapy alone or in combination with magnetic hyperthermia in prostate cancer models. Therefore, this research aims to assess the synergistic effects of electrochemotherapy (employing electroporation and the chemotherapeutic drugs bleomycin and cisplatin) combined with electromagnetic hyperthermia (utilizing SPION’s) on the DU-145/GFP cell line in vitro.

## 2. Materials and Methods (Figure S1)

### 2.1. Cell Culture 

The DU-145/GFP cell line was obtained from Anticancer, Inc. (San Diego, CA, USA) The cells were cultured in RPMI 1640 growth medium (Sigma Life Science Cat. No. R8758, Darmstadt, Alemania), supplemented with 10% FBS (By productos, SF-FB), penicillin (100 U/mL), and streptomycin (100 µg/mL) (Gibco Cat. No. 15140-122, New York, NY, USA). The RWPE-1 cell line was purchased from the American Type Culture Collection (ATTC, Manassas, VA, USA). The cells were cultured in K-SFM (Gibco Cat. No. 17005, New York, NY, USA ) supplemented with 2.5 µg of epidermal growth factor (EGF) (Gibco Cat. No. 10450-013, New York, NY, USA), 25 mg of bovine pituitary extract (BPE) (Gibco Cat. No. 13028-014, New York, NY, USA), and 1% of penicillin-streptomycin (Gibco Cat. No. 15140-122, New York, NY, USA). The cells were maintained at 37 °C in a humid atmosphere with 5% CO_2_. 

### 2.2. Cytotoxicity Assays with WST-1 

#### 2.2.1. Chemotherapeutic Drugs 

Two chemotherapeutic drugs were used for the experiments: cisplatin (1 mg/mL Aqueous Cytoplatin-50, Cipla, Thane, India) and bleomycin (Bleocel 15 Units, Celon Labs, Hyderabad, India). Bleomycin was dissolved in saline solution to a stock concentration of 2 mg/mL. 

#### 2.2.2. Determination of IC_50_ of Cisplatin, Bleomycin, and Combination of Both Drugs 

DU-145/GFP or RWPE-1 cells were harvested with 0.25% trypsin-EDTA (Gibco Cat. No. 25200056, New York, NY, USA) once they reached 90% confluence in a T-75 flask. A total of 1 × 10^4^ cells from the DU-145/GFP cell line or 2 × 10^4^ cells of the RWPE-1 cell line were seeded in each well of a 96-well plate. After 24 h, different concentrations of cisplatin (10, 30, and 50 µM), bleomycin (10, 30, 50, 100, 150, 200, and 250 µM), and a combination of both (10 µM Bleomycin + 10 µM Cisplatin and 10 µM Bleomycin + 30 µM Cisplatin) were added and incubated for a further 24 and 48 h. The cell viability was measured using the cell proliferation reagent WST-1 (Roche, Cat. No. 11 644 807 001, Mannheim, Germany), adding 20 µL per well and incubating for four hours. Each hour the optical density was read at 450 nm and corrected at 600 nm using a multimode multiplate reader (Synergy HT, Cat. 7091000, Biotek Instruments Inc., Winooski, VT, USA). 

#### 2.2.3. Electrochemotherapy In Vitro Standardization 

To set up the electroporation conditions that keep at least 90% cell viability in the DU-145/GFP cell line, different electric fields (900, 1000, 1100, 1200, and 1300 V/cm) were applied alone or in combination with cisplatin (20 µM). A total of 1 × 10^5^ cells per well in a 1 mL final volume were seeded in a 24-well plate and 8 monopolar square wave pulses of 100 µs duration were delivered immediately at a repetition frequency of 1 Hz by an electroporation power supply (ELECTROvet EZ, Leroy Biotech, Saint-Orens-de-Gameville, France) using a plate shape contact electrode (8 mm gap) introduced into each well. For the combination of electroporation and 20 µM cisplatin, 1 × 10^5^ cells were collected in 500 µL of RPMI 1640 medium and 500 µL of cisplatin treatment was added, then the cells were transferred to a 24-well plate and electroporation was applied. After electroporation, 100 µL of cell suspension were seeded per triplicate in a 96-well plate and incubated for 24 and 48 h. Cell viability was measured using the cell proliferation reagent WST-1, adding 10 µL per well and incubating for four hours. The optical density at 450 nm and 600 nm was determined each hour using a microplate reader (Synergy HT, Cat. 7091000, Biotek Instruments Inc., Winooski, VT, USA). 

#### 2.2.4. Determination of IC_50_ of Cisplatin and Bleomycin with Electrochemotherapy 

For this assay, 1 × 10^5^ cells per well (1 mL) were seeded in a 24-well plate and only electroporated as control group. In the case of electroporation combined with cisplatin and bleomycin, 2 × 10^5^ cells were resuspended after trypsinization in two mL of RPMI 1640 medium with different concentrations of cisplatin (10, 20, and 30 µM) and bleomycin (1, 5, and 10 µM). One mL of this suspension was placed in a 24-well plate and 100 µL of the remaining volume was placed in a 96-well plate (these are the control groups of the drugs without electroporation). After seeding the cells in a 24-well plate, the electroporation was delivered to all experimental groups as described above. Once the electroporation was completed, 100 µL from each group were transferred per triplicate to a 96-well plate and incubated for 24 and 48 h. Cell viability was measured using the cell proliferation reagent WST-1, adding 20 µL per well and incubating for four hours. The optical density at 450 nm and 600 nm was determined each hour using a microplate reader (Synergy HT, Cat. 7091000, Biotek Instruments Inc., Winooski, VT, USA). The same procedure was followed for the prostate epithelial cell line RWPE-1, with the only difference being that 2 × 10^5^ cells per well were seeded in a 24-well plate and 4 × 10^5^ cells per well were resuspended in the different treatments with cisplatin and bleomycin. 

#### 2.2.5. SPION’s Heating Capability and Cytotoxicity 

Hyperthermia assays were performed using a ferrofluid of uncoated superparamagnetic iron oxide nanoparticles (SPIONs) synthesized by the co-precipitation method. The SPIONs obtained were 13 nm of average diameter, 70 emu/g of magnetic saturation, and 225 K of blocking temperature [31].

The optimal concentrations of SPION used for hyperthermia assays were those that allowed an elevation of at least 1 °C/min and that showed low toxicity in prostate cancer-derived cell lines. The first assay consisted of SPION’s capability to elevate the temperature by irradiating different concentrations (0.5, 1, 2, and 3 mg/mL) of SPIONs with an electromagnetic field at 460 kHz of heating frequency (f) and an amplitude (H = 20 kA/m) for 5 min. Temperature was measured with a fiber optic probe. The second assay consisted of a cytotoxicity evaluation of SPIONs in DU-145/GFP and RWPE-1 cell lines. A total of 3 × 10^4^ cells per well in 250 µL of RPMI 1640 medium was seeded onto a 48-well plate. After 24 h of incubation, 0.5, 1, 2, and 3 mg/mL of SPIONs were added and the cells were incubated for a further 24 h. Thereafter, the medium with SPIONs was discarded and cells were washed with D-PBS; finally, 100 µL of fresh medium was added. Cell viability was measured using the cell proliferation reagent WST-1, adding 10 µL per well and incubating for four hours. Each hour, the optical density at 450 nm with correction at 600 nm was determined using a microplate reader (Synergy HT, Cat. 7091000, Biotek Instruments Inc., Winooski, VT, USA). Assays with the RWPE-1 cell line followed the same procedure. 

#### 2.2.6. Electromagnetic Hyperthermia Standardization In Vitro 

The most suitable time for electromagnetic hyperthermia was set using the DU-145/GFP cell line and considering that the irradiation time of the treatment should at least reduce 50% of cell viability. For this assay, 3 × 10^4^ cells per well in 250 µL of RPMI 1640 medium were seeded onto a 48-well plate, then 250 µL of the corresponding treatment was added (RPMI 1640 medium and 1 mg/mL of SPIONs) and incubated. In the case of electromagnetic hyperthermia treatment, 9 × 10^4^ cells (it is important to clarify that cells were immediately used after trypsinization when they were still suspended in the medium) were collected in 2 mL microtubes in a volume of 250 µL of RPMI 1640 medium with each one corresponding to a different time of irradiation (5, 10, and 15 min), and then 250 µL of 1.5 mg/mL of SPIONs was added into the tube and it was incubated at 37 °C for 20 min. During the electromagnetic hyperthermia treatment, this temperature was maintained using a dry bath. Each tube was irradiated with an electromagnetic field at 460 kHz of heating frequency (f) and an amplitude (H = 20 kA/m) for 5 min until it reached 43 °C; this temperature was kept at varying H from 12 to 20 kA/m for 5, 10, and 15 min. Temperature was measured using a fiber optic probe. After the electromagnetic hyperthermia treatment was applied to each microtube, 1 mL of RPMI 1640 medium was added to reach a final concentration of 1 mg/mL of SPIONs and cells were homogenized and transferred (3 × 10^4^ cells in 500 µL) onto the 48-well plate used before for control groups and incubated. After 24 h, the medium with SPIONs was discarded and cells were washed with D-PBS; finally, 100 µL of fresh medium was added. Cell viability was measured using the cell proliferation reagent WST-1, adding 10 µL per well and incubating for four hours. Each hour the optical density was read at 450 nm and corrected at 600 nm using a multimode microplate reader (Synergy HT, Cat. 7091000, Biotek Instruments Inc., Winooski, VT, USA). 

#### 2.2.7. Electrochemotherapy Combined with Electromagnetic Hyperthermia In Vitro

Once the optimal concentration of SPIONs and the optimal time of electromagnetic hyperthermia treatment were determined, the hyperthermia assay was evaluated in combination with electrochemotherapy in the DU-145/GFP cell line. Treatments were applied to suspended (trypsinized) cells. For the groups of cells without treatment, cells with SPIONs, cells with bleomycin and cisplatin, and cells with SPIONs plus bleomycin and cisplatin, 3 × 10^4^ cells per well were seeded in a volume of 250 µL into a 48-well plate. A total of 250 µL of the corresponding treatment was added: RPMI 1640 medium for the control group, SPIONs at a final concentration of 1 mg/mL, bleomycin (0.5 and 7 µM, IC_50_ of bleomycin in the RWPE-1 and DU-145/GFP cell lines, respectively), cisplatin (27 and 33 µM, IC_50_ of cisplatin in the RWPE-1 and DU-145/GFP cell lines, respectively), bleomycin (0.5 and 7 µM) plus SPIONs (1 mg/mL), and cisplatin (27 and 33 µM) plus SPIONs (1 mg/mL).

To all of the above groups, the electroporation variable was added. For this, 9 × 10^4^ cells were collected in 750 µL of RPMI medium and 750 µL of the above treatments were added. Then, using plate-shaped electrodes with an 8 mm gap between them, 8 pulses of monopolar square-wave of 100 µs at 1000 V/cm with a repetition frequency of 1 Hz were applied using an electroporator (ELECTROvet EZ, Leroy Biotech), cells were homogenized, and 3 × 10^4^ cells/500 µL were transferred to the 48-well plate used for the control groups and incubated for 24 h.

For the experimental groups subjected to hyperthermia, 9 × 10^4^ cells were collected in 250 µL of RPMI medium in a 2 mL microtube and 250 µL of the corresponding treatment was added: (a) 1.5 mg/mL of SPIONs for the hyperthermia group, and (b) hyperthermia plus electroporation, and (c) 1.5 mg/mL of SPIONs plus bleomycin (0.5 and 7 µM), and (d) 1.5 mg/mL of SPIONs plus cisplatin (27 and 33 µM) for the hyperthermia plus drug groups and the hyperthermia plus drug plus electroporation groups. In the case of the electroporation groups, the corresponding volume was transferred from the microtube to a 24-well plate and 8 pulses of monopolar square wave of 100 µs at 1000 V/cm with a repetition frequency of 1 Hz were applied using a plate-shaped electrode with an 8 mm gap between them and an electroporator (ELECTROvet EZ, Leroy Biotech); then, they were returned to the 2 mL microtube.

All hyperthermia experimental groups were incubated at 37 °C for 20 min and with the help of a dry bath were maintained at 37 °C during the hyperthermia process. Each tube was irradiated with an electromagnetic field at a heating frequency (f) of 460 kHz and an amplitude (H) of 20 kA/m for 10 min; in the first 5 min, a temperature of 43 °C was reached and this was maintained for 5 min by modifying the amplitude conditions. The temperature was measured using a fiber optic probe.

Once hyperthermia was induced in all experimental groups, 1 mL of RPMI 1640 medium was added to each tube to obtain a final concentration of 1 mg/mL of SPIONs, the cells were homogenized, and 3 × 10^4^ cells/500 µL were transferred to a 48-well plate and incubated. After 24 h, the old medium with SPIONs was discarded and cells were washed with D-PBS; finally, 100 µL of fresh medium were added. Cell viability was measured using the cell proliferation reagent WST-1, adding 10 µL per well and incubating for four hours. Each hour the optical density was read at 450 nm and corrected at 600 nm using a microplate reader (Synergy, Biotek). The same procedure was followed for the RWPE-1 cell line.

### 2.3. Statistical Analysis

The IC_50_ values of the drugs bleomycin and cisplatin were determined using nonlinear regression with the GraphPad Prism program (version 8.0.1). A statistical analysis of the assays for the different treatments was conducted using one-way and two-way analysis of variance (ANOVA) to analyze the data obtained from treatments with electrochemotherapy and magnetic hyperthermia. Subsequently, the Tukey test was performed for all cases using the GraphPad Prism program (version 8.0.1). The analysis of data obtained from the combination of electrochemotherapy, and electromagnetic hyperthermia was performed by multiple *t*-tests. A statistically significant difference was considered when the *p*-value was <0.05.

A statistically significant difference was considered when the *p*-value was <0.05. Results are presented as the mean ± standard error of at least three replicates.

## 3. Results

### 3.1. Cytotoxic Effect of Cisplatin and Bleomycin on Prostate-Derived Cell Lines

**Figure 1:**
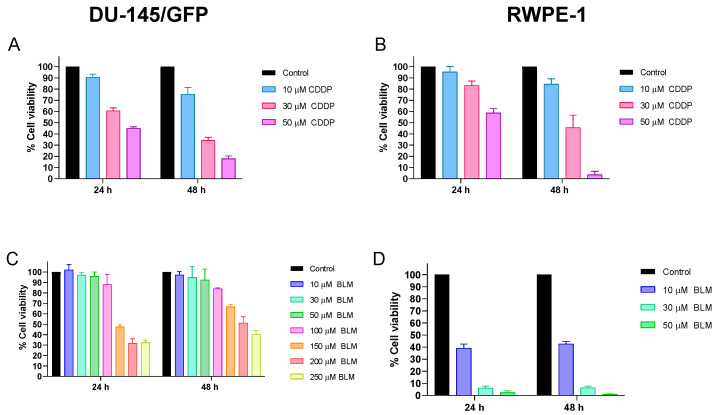
Cytotoxicity evaluation of chemotherapeutics (cisplatin and bleomycin) on prostate-derived cell lines. Cell viability was measured by WST-1 assay and read at OD 450 nm and 600 nm in prostate cancer-derived cell line DU-145/GFP (**A**,**C**) or normal prostate epithelium cell line RWPE-1 (**B**,**D**). IC_50_ was determined using different concentrations of cisplatin (CDDP) (10, 30, and 50 μM), and bleomycin (BLM) (10, 30, 50, 100, 150, 200, and 250 μM). Statistical analysis was performed by two-way ANOVA and Tukey test.

First, the half-maximal inhibitory concentration (IC_50_) of bleomycin and cisplatin was determined to compare this data with further analysis. Cytotoxic assays were performed using different concentrations of bleomycin (10, 30, 50, 100, 150, 200, and 250 µM) and cisplatin (10, 30, and 50 µM) on the prostate cancer-derived cell line DU-145/GFP and on the prostate-derived cell line RWPE-1 as a non-tumorigenic control (Figure 1). Cell viability was measured after 24 and 48 h using the WST-1 test. 

In the DU-145/GFP cell line, the IC_50_ concentrations of cisplatin were 42 and 20 µM at 24 and 48 h, respectively. Conversely, IC_50_ concentrations of cisplatin in the non-tumorigenic cell line RWPE-1 were 59 and 30 µM at 24 and 48 h, respectively. A slight increment can be observed compared to IC_50_ values in DU-145/GFP cells, indicating that the non-tumorigenic cell line is less sensitive than the DU-145/GFP cell line to cisplatin.

In addition, the IC_50_ concentrations of bleomycin in the DU-145/GFP cell line were 157 and 207 µM at 24 and 48 h, respectively, which were significantly higher than cisplatin IC_50_ concentrations. Furthermore, it was observed that the concentrations to reach the IC50 were different at 24 h in the same cell line, suggesting that DU-145/GFP cells have some resistance to bleomycin. However, the IC_50_ concentrations of bleomycin in the RWPE-1 cell line were 8 and 9 µM at 24 and 48 h, respectively, which are markedly lower than the DU-145/GFP cell line’s IC_50_ concentrations, showing that bleomycin has a highly cytotoxic effect over the non-tumorigenic cell line RWPE-1.

### 3.2. Electroporation Enhances the Sensitivity of Prostate-Derived Cell Lines to Chemotherapeutics

#### 3.2.1. Effects of Different Electric Fields on Prostate Cancer-Derived Cell Line DU-145/GFP 

**Figure 2:**
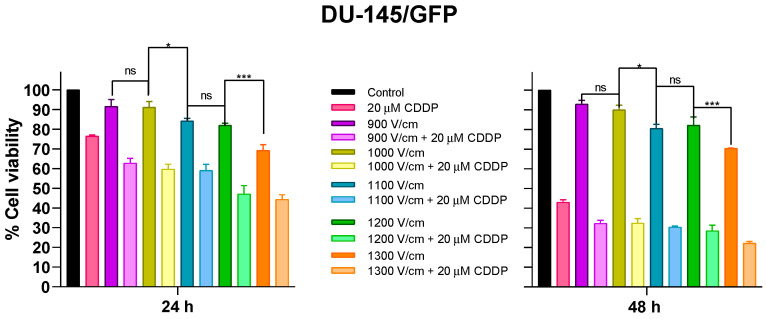
Cytotoxic effects of cisplatin related to the intensity of the electric field in DU-145/GFP cell line. The electroporation was performed alone or combined with 20 μM of cisplatin using different electric fields (900, 1000, 1100, 1200, and 1300 V/cm) in prostate cancer-derived cell line DU-145/GFP. Cell viability was measured by WST-1 assay; OD was determined at 450 nm and 600 nm. Statistical analysis was performed by two-way ANOVA and Tukey test (* *p* < 0.05, *** *p* < 0.001). ns: No statistical significance.

When electroporation is applied to cells, two possible scenarios can occur on plasmatic membranes: (1) the rearrangement of phospholipids changes its permeability, or (2) the disruption of the plasmatic membrane produces cell death. In electrochemotherapy, it is pivotal that the first scenario occurs to improve the plasmatic membrane permeability to chemotherapeutic drugs and increase its intracellular concentration. This scenario can be produced by changing the intensity of the electric field applied to the cells during the electroporation process; to accomplish this specificity, different electric fields were assayed in the DU-145/GFP cell line. For this, eight monopolar square-wave pulses of 100 µs and RF of 1 Hz were applied, and different electric fields (900–1300 V/cm) were tested alone and in combination with 20 µM cisplatin (half the value of the IC_50_ of cisplatin in DU-145/GFP), expecting that electroporation could increase the cytotoxic effect of cisplatin by up to 50% (Figure 2).

It was found that an appropriate intensity of electric field for in vitro assays is 1000 V/cm, as it reduces cell viability by 10% and significantly increases the cytotoxic effect of cisplatin compared to cisplatin control without electroporation (*p* < 0.05). Additionally, there were no significant differences between cell viability percentages from the electric field 1100 V/cm in combination with cisplatin at 24 h. However, there were significant differences between the electric fields 1100 V/cm and 1000 V/cm alone on cell viability at 24 and 48 h. Therefore, the electric field used in future electrochemotherapy assays is 1000 V/cm.

#### 3.2.2. Cytotoxic Effect of Electrochemotherapy with Cisplatin and Bleomycin on Prostate-Derived Cell Lines

**Figure 3:**
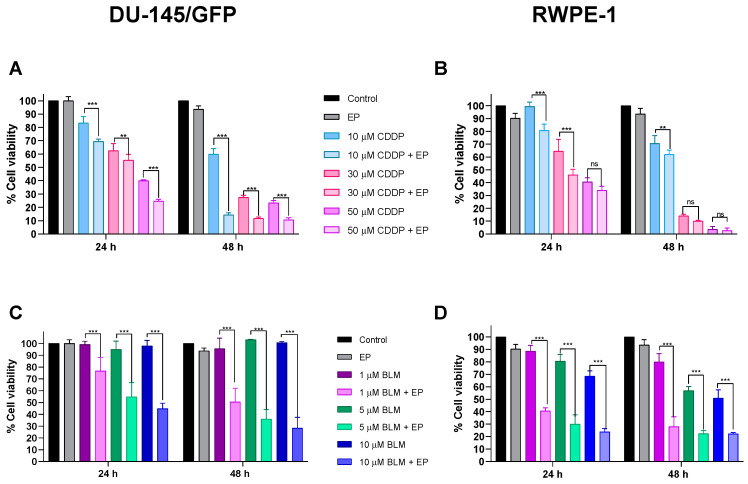
Cytotoxicity evaluation of electrochemotherapy in prostate-derived cell lines. The cell lines DU-145/GFP (**A**,**C**) and RWPE-1 (**B**,**D**) were electroporated by applying 1000 V/cm. IC50 was determined using different concentrations of cisplatin (CDDP) (10, 30, and 50 μM) and bleomycin (BLM) (1, 5, and 10 μM) combined with electroporation (EP). Cell viability was measured by WST-1 assay and read at OD 450 nm and 600 nm. Statistical analysis was performed by two-way ANOVA and Tukey test (** *p* < 0.01, *** *p* < 0.001). ns: No statistical significance.

Using the electric field intensity of 1000 V/cm, IC_50_ concentrations of cisplatin and bleomycin in the prostate-derived cell lines DU-145/GFP and RWPE-1 were determined by applying electroporation (eight monopolar square-wave pulses of 100 µs and RF of 1 Hz) alone and in combination with different concentrations of cisplatin (10, 30, and 50 µM) and bleomycin (1, 5, and 10 µM) and evaluating cytotoxicity at 24 and 48 h using the WST-1 test (Figure 3).

Electroporation increased its cytotoxic effect in the DU-145/GFP cell line, 1.27 and 8.45 times at 24 and 48 h, respectively (IC_50_ concentrations were 33 and 2 µM at 24 and 48 h, respectively), implying that electrochemotherapy can reduce the cisplatin dosage and produce an equal or even greater effect than the standard dosage used in chemotherapy treatment. A similar effect can be observed in the RWPE-1 cell line at 24 h with an increment of the cytotoxic effect of cisplatin of 2.17 times (IC_50_ concentrations were 27.04 and 12.21 µM at 24 and 48 h, respectively); however, at 48 h this increase remains constant at 2.36 times, which is reduced compared to the increment of the cytotoxic effect in the DU-145/GFP cell line. It is important to note that, after 48 h of the treatment in the non-tumorigenic cell line, using higher concentrations of cisplatin (30 and 50 µM) has no significant effect on increasing the cytotoxic effect of the drug using electroporation, this could indicate that the use of lower concentrations of cisplatin (<10 µM) can protect the healthy tissue surrounding the tumor using electrochemotherapy.

The increase in the cytotoxicity of bleomycin due to electroporation is greater in both cell lines compared to cisplatin. The IC_50_ concentrations in the DU-145/GFP cell line were 7.105 and 1.883 µM at 24 and 48 h, respectively, observing an increase of 22 and 110 times. This confirms that the resistance of the drug observed previously was due to its null permeability to the plasmatic cell membrane. As observed in cisplatin, electrochemotherapy is more effective using low concentrations of bleomycin. 

As expected, electrochemotherapy with bleomycin in the RWPE-1 cell line has more cytotoxicity over the cells compared to the DU-145/GFP cell line because IC_50_ concentrations were 0.55 and 0.066 µM at 24 and 48 h, respectively, indicating an increase of 15 and 133 times.

### 3.3. Irradiation Time of SPIONs Influences Inhibition of Cell Proliferation of Prostate Cancer Cells

#### 3.3.1. Determination of Optimal Concentration of SPIONs for Electromagnetic Hyperthermia on Prostate-Derived Cell Lines

**Figure 4:**
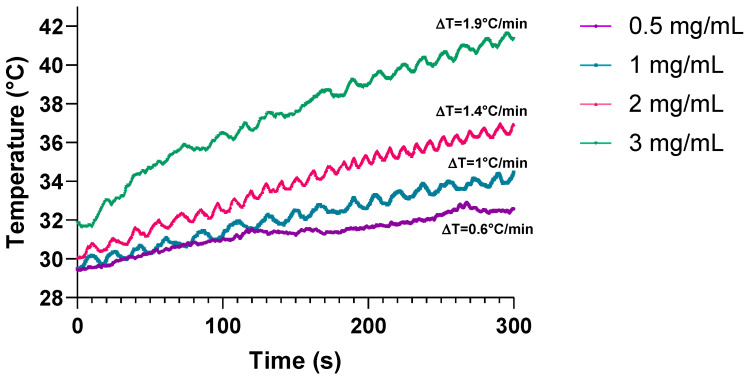
Heating capacity of superparamagnetic iron oxide nanoparticles (SPIONs). Four different concentrations of SPIONs (0.5 mg/mL, 1 mg/mL, 2 mg/mL, and 3 mg/mL) were irradiated with an electromagnetic field at a heating frequency (f) of 460 kHz and an amplitude (H) of 20 kA/m for five min. The temperature was measured with a fiber optic thermometer.

The following assays required the determination of the optimal concentration of SPIONs, which should be a concentration with low toxicity to the cells and should increase at least 1 °C/min when the ferrofluid is irradiated with an electromagnetic field. The optimal concentration was determined by evaluating the heating capacity of different concentrations of SPIONs in a ferrofluid (0.5, 1, 2, and 3 mg/mL) which was irradiated with an electromagnetic field at a heating frequency (f = 460 kHz) and an amplitude (H = 20 kA/m) for 5 min. It was found that by irradiating concentrations of 1, 2, and 3 mg/mL the temperature increases at least 1 °C/min (Figure 4).

**Figure 5:**
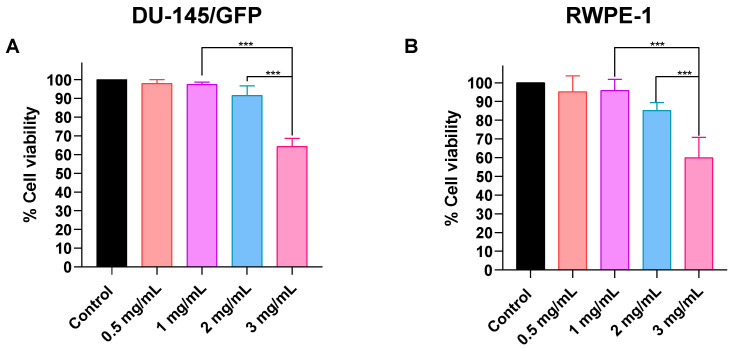
Cytotoxicity evaluation of superparamagnetic iron oxide nanoparticles (SPIONs) in prostate-derived cell lines. Different concentrations of SPIONs were added (0.5, 1, 2, and 3 mg/mL) and cell viability was measured by WST-1 assay and read at OD 450 nm and 600 nm in prostate cancer-derived cell line DU-145/GFP (**A**) and normal prostate epithelium cell line RWPE-1 (**B**). Statistical analysis was performed by two-way ANOVA and Tukey test (*** *p* < 0.001).

Afterward, SPIONs’ cytotoxicity was evaluated using the same concentrations in both tumorigenic and non-tumorigenic prostate-derived cell lines. Figure 5 shows that 0.5, 1, and 2 mg/mL concentrations have no significant effect on cell proliferation in both cell lines. However, it can be observed that the RWPE-1 cell line is slightly more sensitive to SPIONs. Considering these results the optimal concentration is 1 mg/mL, because it elevates the temperature 1 °C/min and reduces the cell viability less than 5% in both cell lines.

#### 3.3.2. Effects of Different Irradiation Times on Cell Proliferation in Prostate Cancer-Derived Cell Line DU-145/GFP

**Figure 6:**
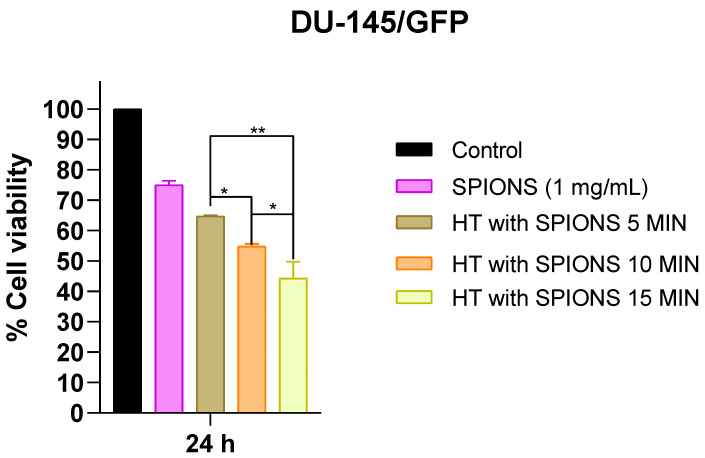
Cytotoxic effect of electromagnetic hyperthermia (HT) related to irradiation time in a prostate-cancer-derived cell line. A concentration of SPIONs (1 mg/mL) was added and then irradiated with an electromagnetic field at a heating frequency (f) of 460 kHz and an amplitude (H) of 20 kA/m for 5 min until reaching 43 °C and it was maintained for 5, 10, and 15 min more in prostate-cancer-derived cell line DU-145/GFP. Cell viability was measured by WST-1 assay and read at OD 450 nm and 600 nm. Statistical analysis was performed by one-way ANOVA and Tukey test (* *p* < 0.05, ** *p* < 0.01).

The time of irradiation to maintain electromagnetic hyperthermia in the cells also influences the results of the next assays; hence, it is necessary to establish the adequate time of electromagnetic hyperthermia. To evaluate this, the DU-145/GFP was incubated in 0.5 mL of the ferrofluid at a concentration of 1 mg/mL of SPIONs for 20 min and then the cells were irradiated with an electromagnetic field at a heating frequency (f = 460 kHz) and an amplitude (H = 20 kA/m) for 5 min until reaching 43 °C and it was maintained for another 5, 10, and 15 min at varying H (12–20 kA/m). Figure 6 shows a proportional relation between electromagnetic hyperthermia time and cytotoxicity. For further assays, the cells were exposed to the lowest time of irradiation (5 min), because it would be combined with electrochemotherapy.

### 3.4. Electrochemotherapy and Electromagnetic Hyperthermia Significantly Decrease Cell Proliferation of Prostate-Cancer Cells

#### 3.4.1. Electroporation Increases the Effectiveness of Electromagnetic Hyperthermia in Prostate-Derived Cell Lines

**Figure 7:**
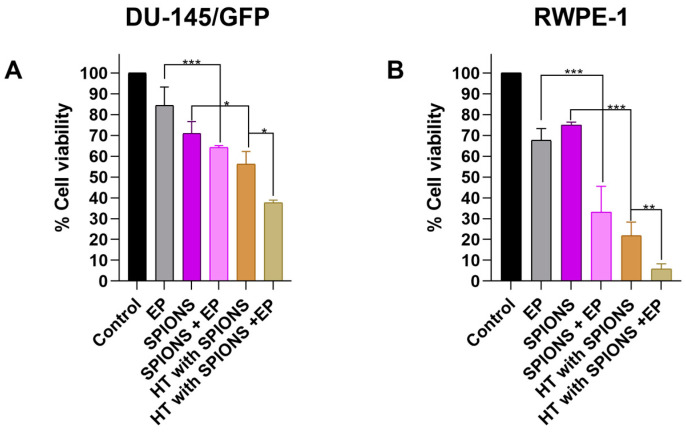
Cytotoxicity evaluation of electromagnetic hyperthermia (HT) combined with electroporation (EP) in prostate-derived cell lines. A concentration of SPIONs (1 mg/mL) was added to cell lines DU-145/GFP (**A**) and RWPE-1 (**B**) and then electroporation was applied (1000 V/cm). Afterward, it was then irradiated with an electromagnetic field at a heating frequency (f) of 460 kHz and an amplitude (H) of 20 kA/m for 5 min until reaching a temperature of 43 °C and then maintained for a further 5 min. Cell viability was measured by WST-1 assay and read at OD 450 nm and 600 nm. Statistical analysis was performed by one-way ANOVA and Tukey test (* *p* < 0.05, ** *p* < 0.01, *** *p* < 0.001).

The diameter of the SPIONs used in this study was 13 nm [31]. Normally, these nanoparticles enter the cell via clathrin-mediated endocytosis. Nonetheless, the use of electroporation could increase the number of nanoparticles in the interior of the cell, therefore increasing the cytotoxic effect of electromagnetic hyperthermia. The latter was proved in this study by applying electromagnetic hyperthermia alone and in combination with electroporation in both tumorigenic (DU-145/GFP) and non-tumorigenic (RWPE-1) prostate-derived cell lines. The results are shown in Figure 7, where a significant increase in the cytotoxic effect of electromagnetic hyperthermia can be observed by adding electroporation before the irradiation of the cells with an electromagnetic field. This effect is greater in the RWPE-1 cell line. 

However, when the cytotoxicity of the SPIONs alone is compared with the addition of electroporation to the SPIONs in the DU-145/GFP cell line, a slight difference in cell proliferation is observed, indicating that the increase in the intracellular concentration of SPIONs does not have a significant increase in the cytotoxic effect of the SPIONs, but it is significant when the cells are irradiated with the electromagnetic field after the electroporation. In the non-tumorigenic cell line RWPE-1, a significant increase in the cytotoxic effect of the SPIONs is observed by adding only electroporation, suggesting that the increase in the cytotoxic effect is due to both the increase in the intracellular SPION concentration and the irradiation with an electromagnetic field in this cell line, indicating that the non-tumorigenic cell line RWPE-1 is more sensitive to electromagnetic hyperthermia treatment than the tumorigenic cell line DU-145/GFP.

On the other hand, the treatment with electromagnetic hyperthermia and the combination of electroporation and SPIONs have similar effects on cell proliferation in both cell lines suggesting that applying electromagnetic hyperthermia for 5 min has a similar effect to the increase in the intracellular concentration by applying electroporation in in vitro conditions; however, this also suggests that combining electroporation with electromagnetic hyperthermia allows for a reduction in the time of exposure to electromagnetic hyperthermia, because the effect on cell proliferation observed with the addition of electroporation to electromagnetic hyperthermia is similar to exposing cells to electromagnetic hyperthermia for 15 min (Figure 6). 

#### 3.4.2. Effect of Electrochemotherapy with Bleomycin and Cisplatin in Combination with Electromagnetic Hyperthermia on Prostate-Derived Cell Lines

**Figure 8:**
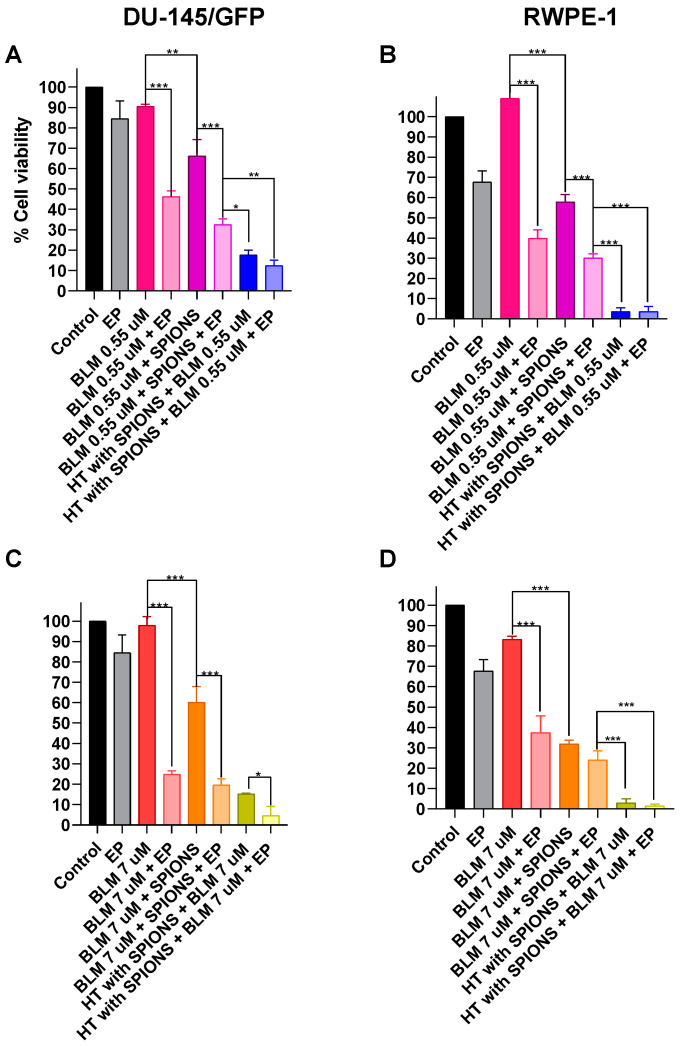
Cytotoxicity evaluation of electromagnetic hyperthermia (HT) combined with chemotherapy and electrochemotherapy with bleomycin (BLM) in prostate-derived cell lines. A concentration of SPIONs (1 mg/mL) was added in combination with bleomycin 0.55 µM and 7 µM to the DU-145GFP cell line (**A**,**C**) and RWPE-1 cells (**B**,**D**); then, electroporation was applied (1000 V/cm) and afterward, it was irradiated with an electromagnetic field at a heating frequency (f) of 460 kHz and an amplitude (H) of 20 kA/m for 5 min until a temperature of 43 °C was reached and then it was maintained for 5 more minutes. Cell viability was measured by WST-1 assay and read at OD 450 nm and 600 nm. Statistical analysis was performed by multiple *t*-tests (* *p* < 0.05, ** *p* < 0.01, *** *p* < 0.001).

**Figure 9:**
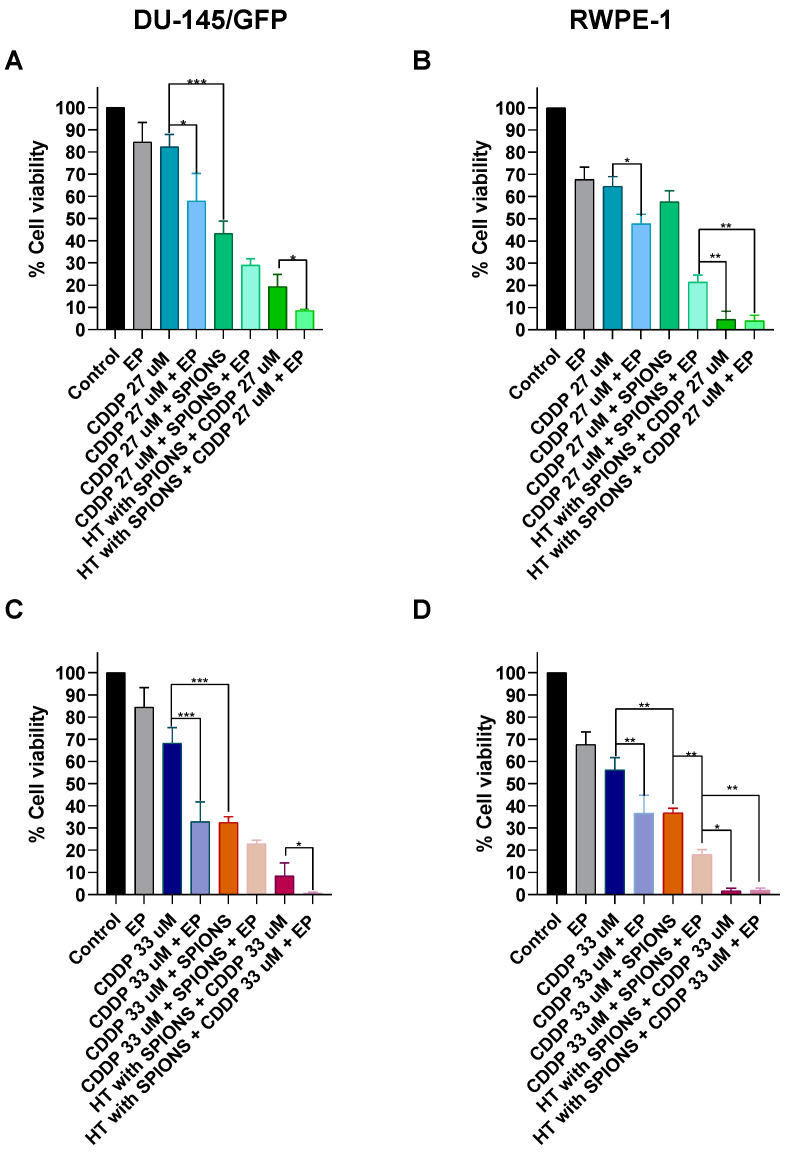
Cytotoxicity evaluation of electromagnetic hyperthermia (HT) combined with chemotherapy and electrochemotherapy with cisplatin (CDDP) in prostate-derived cell lines. A total of 1 mg/mL of SPIONs was added in combination with cisplatin 27 µM and 33 µM to cell lines DU-145/GFP (**A**,**C**) and RWPE-1 (**B**,**D**), then electroporation was applied (1000 V/cm) and afterward, it was irradiated with an electromagnetic field at a heating frequency (f) of 460 kHz and an amplitude (H) of 20 kA/m for 5 min until a temperature of 43 °C was reached and it was maintained for 5 more minutes. Cell viability was measured by WST-1 assay and read at OD 450 nm and 600 nm. Statistical analysis was performed by multiple *t*-tests (* *p* < 0.05, ** *p* < 0.01, *** *p* < 0.001).

Finally, the electromagnetic hyperthermia was combined with the addition of the chemotherapeutic drugs bleomycin (0.55 and 7 µM, which are the IC_50_ determined for RWPE-1 and DU-145/GFP cell lines, respectively) and cisplatin (27 and 33 µM, which are the IC_50_ determined for RWPE-1 and DU-145/GFP cell lines, respectively) and electroporation (electrochemotherapy) (Figure 8 and Figure 9). 

The addition of the two concentrations of bleomycin to the electromagnetic hyperthermia treatment shows an increase in the cytotoxicity compared to control treatments (bleomycin and electromagnetic hyperthermia alone), suggesting that hyperthermia treatment sensibilizes cells to the effects of the drug; moreover, when lower concentrations are used (0.55 µM) this increase is greater than the observed when the intracellular concentration of the drug and SPIONs are increased by the application of electroporation. The latter effect is observed in both concentrations of bleomycin in the RWPE-1 cell line.

In the tumorigenic cell line, DU-145/GFP, when using the higher concentration of bleomycin (7 µM), a significant increase in the cytotoxic effect of electromagnetic hyperthermia combined with electroporation can be observed. This effect is only observed using this concentration and it is not observed in the RWPE-1 cell line, due to the high cytotoxicity of both treatments in this cell line (Figure 8).

Conversely, the addition of both concentrations of the drug cisplatin to the electromagnetic hyperthermia treatment increases the cytotoxic effect of electromagnetic hyperthermia compared to control treatments (cisplatin and electromagnetic hyperthermia alone) in both cell lines as seen before with bleomycin. When electroporation is applied to increase the intracellular concentration of SPIONs and cisplatin, it has an effect on cells that is similar to the combination of the electromagnetic hyperthermia treatment with both combinations of cisplatin. This effect is significant in the RWPE-1 cell line. 

The combination of electrochemotherapy (electroporation and cisplatin) and electromagnetic hyperthermia increases significantly the cytotoxic effect compared to the electromagnetic hyperthermia combined with cisplatin alone, suggesting an additive effect of the rise in the intracellular concentration of SPIONs and cisplatin with the sensibilization of the cells to the cisplatin favored by the electromagnetic hyperthermia treatment. This effect cannot be observed in the RWPE-1 cell line due to the high cytotoxicity of both treatments in this cell line (Figure 9). 

## 4. Discussion

Chemotherapy stands as the primary modality for managing advanced stages of prostate cancer characterized by androgen independence. Nonetheless, its non-specific nature induces side effects that constrain its utility. Adjuvant approaches such as electrochemotherapy seek to diminish the dosage of chemotherapeutic agents like bleomycin and cisplatin, concomitantly augmenting their cytotoxicity. This augmentation enhances therapeutic efficacy while mitigating adverse effects. Electrochemotherapy harnesses electroporation, facilitating the entrance of molecules such as drugs and nanoparticles. Specifically, superparamagnetic iron oxide nanoparticles (SPIONs) are used to elicit magnetic hyperthermia, thereby affecting tumor cell proliferation. It is hypothesized that synergistically, when combined with electroporation, an increased nanoparticle influx into the cell is anticipated, heightening the cytotoxicity of hyperthermia. The principal purpose of this research is to assess the impact of combining electrochemotherapy with electromagnetic hyperthermia on cellular proliferation within prostate-derived-cell lines.

Regarding the effect of the drugs alone on both cell lines, it was found that IC_50_ concentrations for cisplatin were higher in the RWPE-1 cell line (58.85 and 28.90 µM at 24 and 48 h, respectively) than in the DU-145/GFP cell line (42.27 µM and 19.88 µM at 24 and 48 h, respectively) indicating that the tumorigenic cell line is more sensitive to the drug. The drug cisplatin has low permeability to the cell membrane and enters the cell through passive diffusion; however, a possible active transport has also been reported through transporters analogous to MDR1 (multidrug resistance protein 1), and CTR1 (copper transporter 1), which is hypothesized to introduce the drug into the cytoplasm via pinocytosis and vesicular transport [32,33]. Cells with a higher expression of CTR1 may exhibit an increased accumulation of cisplatin [34], which can be a possible explanation to this difference in the IC_50_ concentrations in both cell lines. Although cisplatin is less cytotoxic in the non-tumorigenic cell line, cell death is still observed, especially at concentrations of 30 and 50 µM after 48 h of treatment, which is consistent with findings reported by Moreno et al. [35] and Ormerod et al. [36]. They reported that cisplatin exhibits a dual mechanism of cell death depending on the administered concentrations; at high concentrations (18–50 µM), the cell death mechanism is faster and associated with significant side effects, indicating the importance of developing strategies to reduce the dosage and consequently its side effects on healthy tissues. 

In contrast, IC_50_ concentrations were greater than cisplatin in the DU-145/GFP cell line. Bleomycin, due to its physicochemical properties, has null permeability to the cell membrane, thereby preventing its entry through diffusion and requiring a membrane-associated protein for entry via endocytosis [37]. In vitro studies have demonstrated that only 0.1% of the total administered drug enters the cells [38] and the use of this chemotherapeutic agent is limited due to its high toxicity in the lungs and skin [39,40], so it might be highly cytotoxic in normal prostate epithelial tissue. Some studies mention that keratinocytes are particularly sensitive to bleomycin, as its treatment in in vitro studies results in apoptosis [40], which could explain why RWPE-1 (keratinocyte-derived cell line) is highly sensitive to the drug.

Consequently, for electroporation conditions, an electric field of 1000 V/cm was chosen, as it reduces cell viability by 10%. These results correlate with those of Kielbik et al. [41], who reported that in the DU-145 cell line, an electric field intensity of 1000 V/cm provides relatively high cellular permeability (~70% permeated cells) without significantly reducing cell viability (~90%). After applying electroporation, the IC_50_ concentrations of both drugs were reduced in both cell lines. In the DU-145/GFP cell line, the cytotoxic effect of cisplatin increased 1.27 and 8.45 times at 24 and 48 h, respectively, demonstrating that electrochemotherapy treatment increases the cytotoxic effect of cisplatin in the DU-145/GFP cell line; however, no studies were found on the effect of electrochemotherapy with cisplatin in vitro in the DU-145 cell line, but similar results have been found in different cell lines. Todorovic et al. [42] reported that the cytotoxic effect of cisplatin increased 2.8 times when combined with electroporation in the murine colorectal carcinoma cell line CMT-93, using a voltage of 1300 V/cm. On the other hand, Saczko et al. [43] reported that when using a voltage of 3000 V/cm in the SKOV-3 cell line (human ovarian carcinoma), the IC_50_ increased 29 times after 24 h; nonetheless, it is important to note that a voltage of 3000 V/cm reduces cell viability to ~60%, as reported in their study, so part of the increase is due to cell death by irreversible electroporation.

Furthermore, in the non-tumorigenic RWPE-1 cell line, electrochemotherapy with cisplatin showed an increase in the cytotoxic effect of 2.17 and 2.36 times, respectively. After 24 h, the IC_50_ of cisplatin in DU-145/GFP and RWPE-1 is similar, implicating that the resistance presented by RWPE-1 to cisplatin could be overcome thanks to electroporation, confirming that the resistance of cisplatin in this cell line was mainly due to the degradation of the CTR1 receptor, as electroporation increased the intracellular concentration of the drug, overcoming the limitation of CTR1 receptor degradation when cisplatin is administered without electroporation [33]. Furthermore, this increase in concentration is related to the mechanism of cell death at the high concentrations of cisplatin [35,36] mentioned earlier. Likewise, it is observed that electrochemotherapy treatment is more effective in DU-145/GFP than in RWPE-1 at 48 h, because the electroporation process is applied only once; thus, cisplatin molecules that remained in the extracellular medium continue to degrade the CTR1 receptor in this cell line, presenting greater resistance after 48 h.

After employing electroporation as a strategy to increase the cytotoxic effect of bleomycin, it was found that its cytotoxic effect increased up to 22 times at 24 h and 110 times at 48 h, confirming that the resistance presented by the DU-145/GFP cell line to the drug was due to its null permeability through the cell membrane. Todorovic et al. [42] reported an increase of up to 500 times in a murine colorectal carcinoma cell line; however, it should be considered that the IC_50_ of this cell line is 0.1 µM, which, compared to the data found in DU-145, does not present great resistance to the drug. According to the results presented in this study, electrochemotherapy with bleomycin is more effective at low concentrations. On the other hand, in the study by Saczko et al. [43], it was found that using a concentration of 300 nM of bleomycin in combination with electroporation (1000 V/cm) reduced viability by 60% at 24 h in an ovarian cancer cell line (SKOV-3). Likewise, it was observed that this effect increases over time, as also observed in this study with the DU-145/GFP cell line. In the RWPE-1 cell line, electrochemotherapy increased the cytotoxic effect of bleomycin 15 and 133 times at 24 and 48 h, respectively, indicating that electrochemotherapy with bleomycin is more effective in the non-tumorigenic cell line, due to the high toxicity that bleomycin presents by itself on the RWPE-1 cell line. However, electrochemotherapy with bleomycin in the tumorigenic cell line allowed a reduction in the dosage of the drug to a lower concentration than the IC_50_ estimated for bleomycin alone in the RWPE-1 cell line, so by applying electroporation only to the tumor tissue, significant side effects on healthy tissues surrounding the tumor would be avoided when the assay is scaled to in vivo and clinical studies.

Moving forward to electromagnetic hyperthermia in this study, it was determined that the appropriate concentration of SPIONs is 1 mg/mL, which is lower than the concentration used in various studies [15,18,31] that used SPIONs with different coatings that modify the properties of the nanoparticles. In this study, uncoated SPIONs were used to avoid adding other variables to the increase in cytotoxic effect in the combination of treatments. The SPION concentration was only determined for in vitro assays, but it is important to consider that this study was designed to extrapolate to in vivo assays where SPIONs would be injected intratumorally, where we can use a concentration of 10 mg/mL in mice, according to the Rosales et al. study, without seeing high toxicity in the mice [44].

In this study, it was found that high concentrations of SPIONs (3 mg/mL) decrease cell viability by approximately 40% in both cell lines (DU-145/GFP and RWPE-1), which differs from what was found in the study by Cervantes et al. [31], where they report that concentrations of up to 4 mg/mL of SPIONs coated with 1,2-benzenediol only decrease cell viability by around 20%. This is because it has been found that uncoated nanoparticles with another material could be more cytotoxic than nanoparticles that are coated because the surface of iron ions of uncoated nanoparticles induces the production of reactive oxygen species (ROS) more efficiently; otherwise, the coating of nanoparticles would function as a barrier that attenuates the production of ROS [45].

Inducing hyperthermia by irradiating a concentration of 1 mg/mL of SPIONs with an electromagnetic field in the DU-145/GFP cell line, it is observed that the cytotoxic effect is significantly increased compared to the cytotoxic effect of SPIONs without irradiation, and this effect is time-dependent. A similar behavior is observed in the study by Asín et al. [46].

On the other hand, the mechanisms of cell death induced by the action of electromagnetic hyperthermia through SPIONs are related to the increasing temperature and the action of SPIONs inside the cell. In this study, it was found that by maintaining hyperthermia at 43 °C for 15 min, cell viability is reduced by more than 50% in the DU-145/GFP cell line. If we compare these results with those reported by other studies in different cell lines such as the study by Cervantes et al. [15], where hyperthermia is induced up to 43 °C for 15 min but using dopamine-coated SPIONs, it is observed that cell viability is only reduced by 20%, indicating that the mechanism of cell death is related to the production of ROS through the enzymatic degradation of SPIONs, since, as mentioned earlier, some coatings can delay the production of ROS, as is the case here. 

Likewise, this effect can be verified by comparing the results of this study with the study by Cervantes et al. [31] where hyperthermia is induced at 43 °C for 15 min using SPIONs coated with 1,2-benzenediol, in which it is observed that cell viability is reduced by more than 80%, indicating that its coating functions as a catalyst for the oxidation reaction for the production of ROS when irradiated with an electromagnetic field, which increases its production and therefore increases its cytotoxic effect. This production of ROS is mainly due to the release of Fe^2+^ ions into the cytosol as a product of the lysosomal enzymatic degradation of SPIONs; these ions participate in the Fenton reaction producing the hydroxyl radical (OH) that damages the cell membrane, proteins, and DNA, activating cell death processes. However, at low concentrations, the cell can activate its antioxidant defense mechanism, explaining the low toxicity of the concentrations of SPIONs used to induce hyperthermia in this study. Sola-Leyva et al. [47] and Du et al. [21] demonstrated that the irradiation of SPIONs with an electromagnetic field increases ROS production compared to ROS production by unirradiated SPIONs, which agrees with the results shown in this study, since electromagnetic hyperthermia induces an increase in cell death compared to the effect of unirradiated SPIONs. Zaso et al. mention that the increase in temperature stimulates the kinetics of the Fenton reaction, so the increase in ROS production when irradiating SPIONs with an electromagnetic field may be due to the increase in temperature.

On the other hand, magnetic nanoparticles, depending on their size and the physicochemical properties of their different coatings, enter the cell through different pathways including passive diffusion, caveolin-mediated endocytosis, clathrin-mediated endocytosis, and receptor-mediated endocytosis [48,49]. SPIONs usually enter the cell through clathrin-mediated endocytosis [50,51]. By combining electroporation with electromagnetic hyperthermia, it was found that electroporation increases the cytotoxic effect of hyperthermia; this increase may be due to an increase in the intracellular concentration of SPIONs, and Du et al. demonstrated that administering a high concentration of SPIONs and irradiating them with an electromagnetic field exacerbates the process of cell death by apoptosis [21], which could explain the significant increase in cytotoxic effect by adding EP to the electromagnetic hyperthermia process. Likewise, the results shown in this study indicate that electroporation can be used to reduce the exposure time to electromagnetic hyperthermia, as it is observed that applying electroporation before inducing hyperthermia and maintaining the hyperthermia temperature (43 °C) for 5 min significantly increases the cytotoxic effect compared to the effect produced by maintaining hyperthermia for 15 min in the DU-145/GFP cell line.

Interestingly, it is observed that maintaining hyperthermia for a short period (5 min) affects cell viability like the effect produced by increasing the intracellular concentration of SPIONs through electroporation in both cell lines (DU-145/GFP and RWPE-1). A possible explanation for this event may be related, once again, to the production of ROS by the action of SPIONs, since increasing the intracellular concentration of SPIONs increases the production of ROS, just as the irradiation of SPIONs with an electromagnetic field does [21], so it can be probable that electroporation increases, to the same extent, the production of ROS, just as inducing hyperthermia at 43 °C for 5 min does. However, experiments to measure ROS production in these conditions are needed to confirm this.

Regarding the combination of hyperthermia with chemotherapy, several studies have reported that hyperthermia enhances the effect of some chemotherapeutic drugs [52,53,54,55]. The results presented in this study indicate that electromagnetic hyperthermia treatment increases the cytotoxic effect of the drugs bleomycin and cisplatin in both cell lines. Specifically, for the drug cisplatin, Alvarez-Berríos et al. reported similar results using the Caco-2 cell line using a cisplatin concentration of 5 µM and 2 mg/mL of dextran carboxymethyl nanoparticles, raising the temperature to 41 °C and maintaining it for 30 min. The authors explain that magnetic hyperthermia increases the activity of cisplatin in colon cancer-derived cells due to the increase in membrane fluidity that allows the passive transport of the drug into the cell [53]. Moreover, this study demonstrates that the increase in the intracellular concentration of cisplatin cannot be the only reason why the cytotoxic effect of the drug increases when combined with electromagnetic hyperthermia, since there are significant differences in the cytotoxic effect caused by the increase in the intracellular concentration of cisplatin and SPIONs favored by electroporation, compared to the cytotoxic effect caused by the combination of treatments (hyperthermia + drug) in the RWPE-1 cell line, so it can be inferred that the increase in ROS production is due to the exposure of SPIONs to the electromagnetic field and that the increase in temperature also influence this effect.

Regarding the drug bleomycin, it has been reported that combining bleomycin with heat at a temperature higher than 42 °C enhances its effect and reduces the resistance of several cell lines to the drug [56]. Conversely, Inaoka et al. observed a similar effect in an in vivo model of colon cancer using magnetic nanoparticles, the drug bleomycin, and a vector as a delivery vehicle for the nanoparticles. The researchers reported that the combination of magnetic nanoparticles with bleomycin and irradiation with an alternating magnetic field significantly suppresses tumor growth compared to treatment with the alternating magnetic field and bleomycin alone, which could be due to a synergistic increase in ROS production that exceeds the capacity of the repair of cancer cells [57].

The results presented in this study suggest that combining the drug bleomycin with electromagnetic hyperthermia significantly increases the cytotoxic effect compared to hyperthermia treatment and bleomycin alone. This indicates that a change in membrane fluidity might have occurred, allowing bleomycin to enter the cell as it does with cisplatin [50,52]; however, no evidence was found to demonstrate this.

In the RWPE-1 cell line, it is observed that the increase in the intracellular concentration of bleomycin cannot be the only reason why the cytotoxic effect of the drug increases when combined with electromagnetic hyperthermia, as there are significant differences in the cytotoxic effect caused by the increase in the intracellular concentration of bleomycin and SPIONs thanks to electroporation compared to the cytotoxic effect caused by the combination of treatments (hyperthermia + drug), based on what was reported by Inaoka et al., where the increase in ROS production due to the exposure of SPIONs to the electromagnetic field and the increase in temperature also have an influence. The same effect occurs for the DU-145/GFP cell line at the concentration of 0.55 µM, but this result is not significant for the concentration of 7 µM, so experiments are required to observe ROS production in both conditions and determine if this factor is responsible for this difference.

Additionally, it is observed that under in vitro conditions, electromagnetic hyperthermia and the combination of electromagnetic hyperthermia with chemotherapy and electrochemotherapy are more effective in non-tumor cells (RWPE-1) than in tumor cells (DU-145/GFP); however, it has been reported that there is no apparent difference in heat sensitivity between tumor and non-tumor cells in in vitro models. On the other hand, a selective effect towards cancer cells is observed in in vivo models using temperatures between 40 and 43 °C [54,58].

Finally, the results presented in this study showed that combining electrochemotherapy treatment with electromagnetic hyperthermia significantly increases the cytotoxic effect of cisplatin and bleomycin (7 µM) compared to both therapies separately. One possibility that explains this synergy between treatments is that both processes increase membrane permeability, complementing each other. At the same time, electromagnetic hyperthermia adds more cytotoxicity due to ROS production; however, this combination of treatments needs to be applied in in vivo assays to observe whether the performance of these treatments is different.

## Data Availability

The original contributions presented in the study are included in the article/Supplementary Materials, further inquiries can be directed to the corresponding authors.

## Supplementary Materials

The following supporting information can be downloaded at: https://www.mdpi.com/article/10.3390/pharmaceutics16091109/s1, Figure S1: Schematic diagram of the combination treatment of electrochemotherapy (EQT) and magnetic hyperthermia (HT).
