# Supplementary material for: Synergistic Strategies in Prostate Cancer Therapy: Electrochemotherapy and Electromagnetic Hyperthermia
Source: Pharmaceutics. 2024 Aug 23;16(9):1109. doi: 10.3390/pharmaceutics16091109 (PMC11435295; doi:10.3390/pharmaceutics16091109)
Supplement: Supplementary file 1 [file pharmaceutics-16-01109-s001.zip › pharmaceutics-3134810-supplementary.pdf]

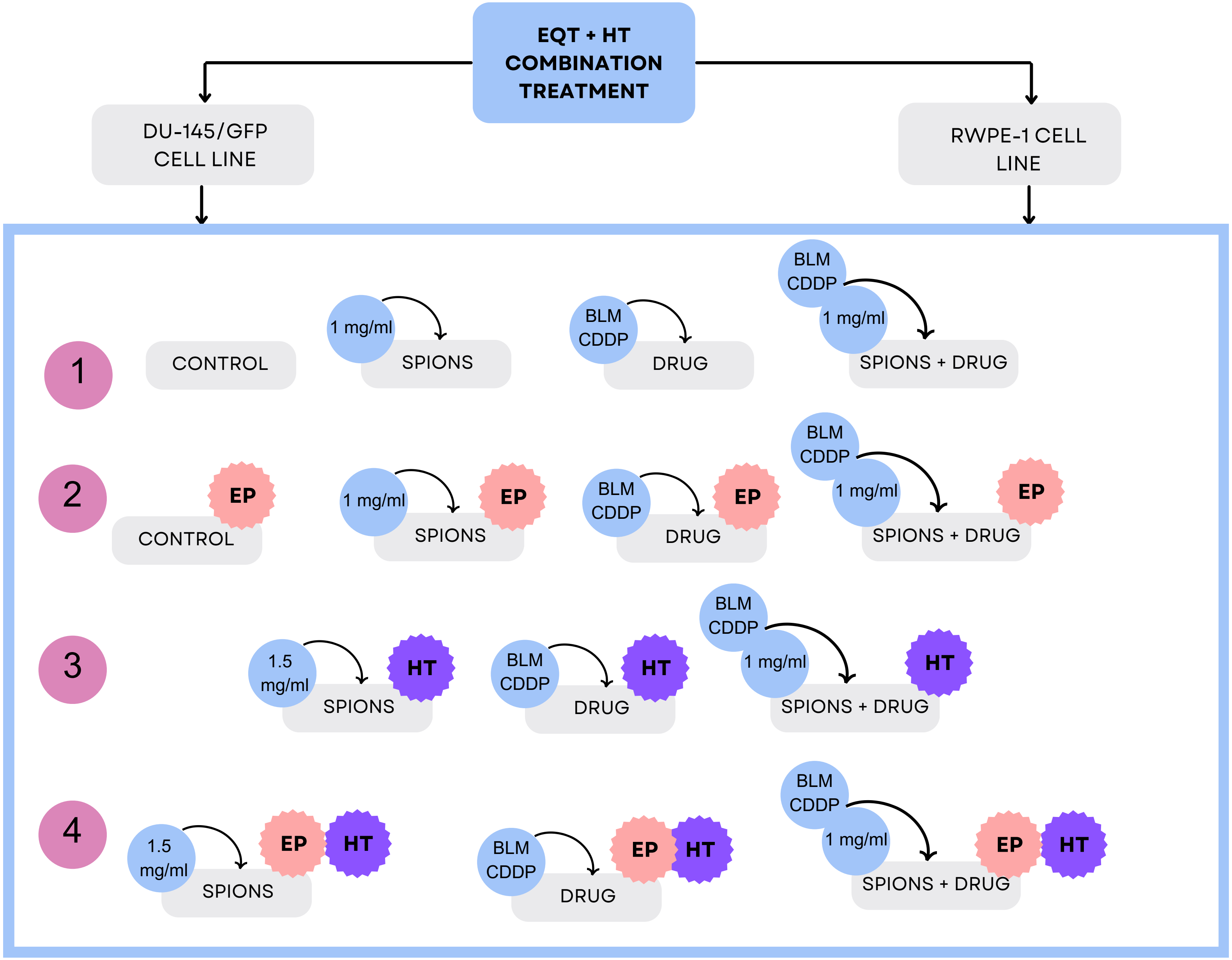

Supplementary figure S1. Schematic diagram of the combination treatment of electrochemotherapy (EQT) and magnetic hyperthermia (HT). 1 mg/ml of SPIONs was added alone and in combination with cisplatin (CDDP, 27 μM and 33 μM) and bleomycin (BLM, 0.5 and 7 μM) to cell lines DU-145/GFP and RWPE-1, then electroporation (EP) was applied (1,000 V/cm) and afterward, it was irradiated with an electromagnetic field at a heating frequency (f) of 460 kHz and an amplitude (H) of 20 kA/m during 5 minutes until a temperature of 43°C was reached and it was maintained for 5 more minutes.
